# Efficacy, Feasibility and Acceptability of a Mediterranean Diet Intervention on Hormonal, Metabolic and Anthropometric Measures in Overweight and Obese Women with Polycystic Ovary Syndrome: Study Protocol

**DOI:** 10.3390/metabo12040311

**Published:** 2022-03-31

**Authors:** Nicole Scannell, Lisa Moran, Evangeline Mantzioris, Stephanie Cowan, Anthony Villani

**Affiliations:** 1 School of Health and Behavioural Sciences, University of the Sunshine Coast, Sunshine Coast, QLD 4556, Australia; nicole.scannell@research.usc.edu.au; 2 Monash Centre for Health Research and Implementation (MCHRI), School of Public Health and Preventive Medicine, Monash University, Melbourne, VIC 3168, Australia; lisa.moran@monash.edu (L.M.); stephanie.cowan@monash.edu (S.C.); 3 UniSA Clinical & Health Sciences, Alliance for Research in Nutrition, Exercise and Activity (ARENA), University of South Australia, Adelaide, SA 5000, Australia; evangeline.mantzioris@unisa.edu.au

**Keywords:** Mediterranean diet, polycystic ovary syndrome, insulin resistance, feasibility, acceptability, behaviour change techniques

## Abstract

Polycystic ovary syndrome (PCOS) is a common endocrine condition in reproductive-aged women associated with metabolic, reproductive and psychological features. Lifestyle modification (diet/physical activity) is considered first-line treatment for PCOS. However, there is limited high-quality evidence to support therapeutic dietary interventions for PCOS beyond general population-based healthy eating guidelines. Adherence to a Mediterranean diet (MedDiet), with or without energy restriction, improves cardiometabolic health in populations including persons with or at high risk of cardiovascular disease and type 2 diabetes. However, there is limited research examining the MedDiet in PCOS. Therefore, this 12 week randomized controlled trial will investigate the efficacy of a MedDiet on cardiometabolic and hormonal parameters and explore its acceptability and feasibility in PCOS. Forty-two overweight and obese women with PCOS (aged 18–45 years) will be randomized to receive dietary advice consistent with Australian Dietary Guidelines or an ad libitum MedDiet intervention. All participants will receive fortnightly counselling to facilitate behaviour change. The primary outcomes will be changes in insulin resistance, glucose, total testosterone and sex hormone-binding globulin. Secondary outcomes include changes in body weight and feasibility and acceptability of the MedDiet intervention. The results of this study will provide further evidence on specific dietary approaches for management of PCOS.

## 1. Introduction

Polycystic ovary syndrome (PCOS) is a common endocrine condition associated with reproductive, metabolic and psychological features [1]. This condition affects women of reproductive age, with a prevalence of 6–18% depending on the diagnostic criteria used and population studied [2,3,4,5]. The European Society for Human Reproduction and Embryology/American Society for Reproductive Medicine (ESHRE/ASRM) international consensus workshop group has expanded the diagnostic criteria for PCOS to the Rotterdam criteria, which is based on women presenting with two of the three following criteria: (1) menstrual irregularity (infrequent menstrual periods) and/or anovulation; (2) clinical and/or biochemical hyperandrogenism; (3) polycystic ovaries based on ultrasound examination [6]. PCOS is underpinned by intrinsic insulin resistance (IR) that, in turn, worsens hormonal and clinical features including hyperandrogenism, hirsutism, oligo-/anovulation and polycystic ovarian morphology [1,7,8]. Infertility is also a clinical feature of PCOS, with up to 75% of these women suffering infertility due to anovulation [9]. Moreover, women with PCOS may experience higher levels of stress, depression and anxiety, which negatively impacts on health-related quality of life and self-efficacy [10,11,12]. For instance, there is evidence to support a higher prevalence of disordered eating behaviours and poor body image amongst women with PCOS, which subsequently contributes to adverse mental health outcomes, including symptoms of depression and anxiety [13,14,15].

Women with PCOS also experience higher levels of weight gain and greater prevalence of overweight and obesity and central obesity, compared to women without PCOS, which exacerbates IR [16,17,18]. As such, this combination of cardiometabolic risk factors substantially increases the risk for cardiovascular disease (CVD), metabolic syndrome and type 2 diabetes mellitus (T2DM) in PCOS [8,19,20]. Weight loss, in turn, improves all clinical features of PCOS [21]. Therefore, lifestyle modifications including adopting a healthy diet and regular physical activity are considered first-line treatment for both weight management (prevention of weight gain, modest weight loss and maintenance of weight loss) and to better manage health outcomes including cardiometabolic disease risk [22]. However, the optimal dietary approach as part of lifestyle management in PCOS remains controversial, with limited high-quality evidence to support any specific dietary approach for PCOS beyond general population-based guidelines from international evidence-based guidelines and recommendations and systematic reviews [23,24,25]. As such, dietary interventions which promote healthy long-term behaviour change, without necessarily focusing on weight loss and caloric restriction, may assist in improving distorted eating behaviours and preserve psychological health [26], which may indeed facilitate adherence and maintenance to longer-term lifestyle and behavioural change. Therefore, defining the optimal dietary approach, either with or without the need for caloric restriction, as a potential therapeutic intervention for the management of cardiometabolic outcomes in PCOS is of clinical interest. 

The MedDiet is a dietary pattern based on the cultural and dietary behaviours of the olive-growing regions of the Mediterranean basin before the mid-1960s [27,28]. Despite being referred to as a ‘diet’, in reality, the MedDiet is more correctly identified as a whole lifestyle approach which also incorporates time-honoured behavioural practices related to food consumption, including harvesting, traditional culinary techniques, frugality and conviviality [27,29]. While there is no single MedDiet, in the research literature, the MedDiet is often described predominately as a plant-based dietary pattern, consistent with a high intake of vegetables, fruits, nuts, legumes, unprocessed cereals, and daily use of extra virgin olive oil (EVOO) incorporated into all meals; moderate consumption of fish, shellfish, fermented dairy products (cheese and yoghurt) and wine (typically during meals); low or infrequent consumption of meat and meat products, processed cereals, sweets, vegetable oils, and butter [30,31]. Being predominately plant-based, the MedDiet is naturally low in saturated fat, and rich in several functional components, including vitamins and minerals, carotenoids, unsaturated fatty acids, and phenolic compounds, depicted by antioxidant and anti-inflammatory properties [32,33,34]. Therefore, the potential mechanistic benefits of the MedDiet on improving clinical features of PCOS may indeed be related to the anti-inflammatory potential of the dietary pattern, reductions in oxidative stress and a higher intake of antioxidants. Moreover, many core foods consistent with a MedDiet pattern (in particular vegetables, legumes, wholegrains, nuts and seeds) naturally have a low glycemic index (GI), which may lower the glycemic response or glycemic load (GL) of the diet [35]. As such, low GI/GL dietary patterns may be beneficial in PCOS through their role in regulating glucose metabolism, and in particular IR [36,37].

Previous clinical trials adopting a Mediterranean diet (MedDiet), with or without caloric restriction, have shown promising results for improving cardiometabolic health parameters in vulnerable populations, including those with or at risk of CVD [38] and T2DM [30,39]. In particular, adherence to a MedDiet affords protection against IR and improves glycemic control, particularly in patients with metabolic perturbations [40,41]. Furthermore, adherence to a MedDiet is inversely associated with central obesity in epidemiological studies and is associated with weight loss in dietary interventions [42,43]. Importantly, many of these benefits are independent of caloric restriction and weight loss due to the large number of functional foods and nutraceuticals present within the dietary pattern.

However, efficacy studies on the MedDiet for the management of PCOS using well-designed and robust clinical trials are scant. Using data from the Australian Longitudinal Study on Women’s Health (ALSWH), Moran et al. [44] reported that women with PCOS were more likely to consume a dietary pattern consistent with principles of a MedDiet, suggesting acceptability of a MedDiet in PCOS. Nevertheless, feasibility and acceptability studies exploring the adherence to a MedDiet are yet to be investigated in women with PCOS and warrant future exploration.

The primary aim of this study is to investigate the efficacy of a MedDiet on hormonal, metabolic and anthropometric measures, without caloric restriction, in overweight and obese women with PCOS. As a secondary aim of the proposed study, we will also explore the acceptability and feasibility of a MedDiet intervention in this same cohort of women.

## 2. Materials and Methods

### 2.1. Study Design, Participants and Recruitment

This study will be a randomized controlled trial (RCT) conducted over 12 weeks, at the University of the Sunshine Coast clinic spaces within the High-Performance Centre. English-speaking participants aged between ≥18 and ≤45 years who are overweight (≥25 kg/m^2^) or obese (≥30 kg/m^2^) with a confirmed diagnosis of PCOS (provided by a medical practitioner) in accordance with the ESRHE/ASRM diagnostic criteria will be eligible to participate. Participants will be recruited throughout Queensland, Australia via local flyers, newspaper advertisements, women’s health and GP clinics, and social media platforms including Facebook, Instagram and Twitter. Recruitment is expected to occur from June 2021 until June 2022. Volunteers who express interest will be screened for eligibility based on the inclusion and exclusion criteria outlined in Table 1. If eligible, volunteers will be invited to participate and will be provided with the research project information sheet and a 4 day food record provided by post or email. At the baseline assessment, participants will be required to provide informed consent prior to the collection of data. 

**Figure 1:**
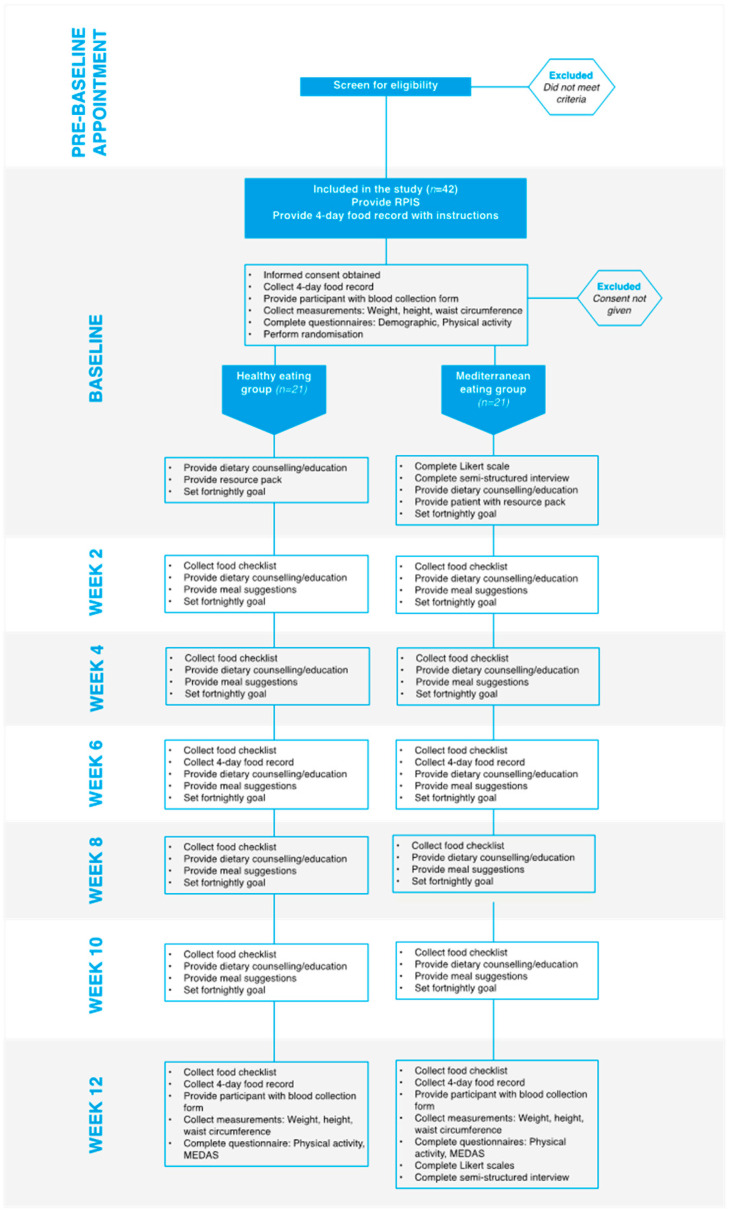
Overview of the study design and schedule.

Participants will be recruited via a rolling recruitment strategy, where they will be randomized to receive either a standard healthy eating (HE) dietary approach consistent with the Australian Dietary Guidelines or a Mediterranean diet (MedDiet) intervention. Allocation will be stratified by BMI (25–29.9 kg/m^2^; ≥30 kg/m^2^) using a random number, computer-generated randomization schedule developed and managed by an independent member of the research team. Participants will attend the clinic for baseline and week 12 assessments and receive fortnightly counselling sessions thereafter for the duration of the study. Participants will be required to attend in-person appointments for the baseline and week 12 assessments, but will be offered the choice of in-person or online consultations for weeks 2, 4, 6, 8 and 10. An overview of the recruitment method, randomization process and study schedule is provided in Figure 1. 

### 2.2. Data Collection

The timeframe for this study will be 15 months, from June 2021 until September 2022. Activities completed in this time include participant recruitment, data collection and data analysis. Outcome measures (primary or secondary) will be assessed at baseline and week 12. The primary outcome measures for this study include biochemical and hormonal parameters including fasting insulin, glucose, total testosterone and sex hormone-binding globulin (SHBG). Secondary outcomes include body weight, waist circumference and the feasibility and adherence to the MedDiet intervention. Adherence to the dietary intervention will be evaluated using dietary checklists, 3 separate 4 day food records (see dietary intervention below) and the MEDAS (see adherence to a MedDiet below).

### 2.3. Biochemical Assessments

After a 12 h overnight fast, participants will be required to provide a venous blood sample collected by a trained phlebotomist at a local Queensland Medical Laboratory (QML) pathology site. Blood samples will be collected at baseline and week 12. Participants will receive a QML blood request form at the baseline and week 12 assessments and instructed to attend a QML pathology collection site immediately following the assessment. Biochemical assessments will include total testosterone, sex hormone-binding globulin, fasting insulin and fasting glucose. Serum concentrations of these biochemical parameters will be collected using blood collection tubes with no additives and allowed to clot at room temperature for 30 min. The homeostatic model assessment (HOMA) will be used as a surrogate measure from which to calculate insulin sensitivity according to the following equation:

Insulin sensitivity = [fasting insulin (mU/L) × fasting glucose (mmol/L)]/22.5 [45].

Analysis of all serum biochemical parameters will be conducted at a local QML pathology laboratory.

### 2.4. Anthrompometry and Body Composition

A calibrated digital scale (AND Weighing; HW-KGL, Melbourne, Australia) will be used to record body mass (without footwear and heavy clothing) to the nearest 0.1 kg. Height will be measured to the nearest 0.1 cm whilst barefoot using a wall-mounted stadiometer (Holtain Limited, Crymych, UK). BMI will be calculated as weight (kg) divided by the square of height (m^2^). Waist circumference will be performed by trained research personnel and measured to the nearest 0.1 cm using a flexible steel tape measure (Lufkin Executive Thinline) at the point midway between the iliac crest and the lower costal border (lower rib) according to standardized protocols. This will be measured in triplicate with the mean of the three measures used for the final analyses. Body weight will be collected at baseline and week 12 and monitored fortnightly throughout the duration of the study. Waist circumference will be assessed at baseline and week 12.

### 2.5. Physical Activity

Physical activity will be monitored using the short version of the International Physical Activity Questionnaire (IPAQ-SF) [46]. IPA-SF quantifies physical activity during the previous seven days and is divided into four categories: vigorous intensity, moderate intensity, walking and sedentary behaviour. Furthermore, intensity, frequency and duration of physical activity are also assessed. Data obtained from the IPAQ-SF will be used to estimate the total amount of physical activity completed in a seven-day period by weighting the reported minutes per week in each of the four physical activity categories by a metabolic equivalent (MET) energy expenditure estimate. MET minutes per week will then be calculated by multiplying the duration (minutes), frequency (days) and MET intensity, and summing across the different categories of physical activity (vigorous, moderate, walking and sedentary behaviour). Participants will be asked to maintain their usual levels of physical activity throughout the intervention period. Physical activity levels will be collected at baseline and week 12.

### 2.6. Dietary Intake

Dietary intake will be recorded using a 4 day food record at baseline, week 6 and week 12 of the study. Prior to each of these assessments, participants will be provided with a 4 day food record and set of written instructions by the study dietitian on how to complete their record. Participants will be asked to record all food and beverage intake for a period of 4 days (3 weekdays and 1 weekend day) using standard household utensils such as standard cup, teaspoon and tablespoon measures, before returning the food record back to the study dietitian at the next appointment. Once returned, all food records will be verbally cross-checked for completeness by the study dietitian, and any ambiguities will be clarified with the participant. All food records (baseline, week 6 and week 12) will be entered into Foodworks, version 10 (Xyris Ltd., Brisbane, Australia) by the study dietitian for a comprehensive nutrient analysis assessment using the Australian Food Composition database. Participants will also be provided with detailed instructions on how to maintain and record their daily dietary checklist. If any deviations are made from the prescribed quantities in the checklist, participants will be instructed to record this. Participants will be instructed to return the dietary checklists each fortnight for the duration of the study and will be used as an additional measure of compliance toward the dietary protocol.

### 2.7. Adherence to the MedDiet Intervention

Adherence to a MedDiet will be assessed at baseline and week 12 using the previously validated 14-item MEDAS, used in the Prevención con Dieta Mediterránea (PREDIMED) study [47]. The MEDAS assesses adherence to a MedDiet according to pre-defined normative criterion cut-offs for the habitual frequency of consumption (pre-defined servings/day or servings/week) of 12 main dietary elements and two food habits related to a traditional MedDiet pattern. Each item in the questionnaire is dichotomously scored as either 0 or 1, producing a maximum score of 14. Greater adherence to a MedDiet is indicated by a higher score. Specifically, a MEDAS score ≥10 is suggestive of high adherence, scores between 6 and 9 indicate moderate adherence, and a score ≤5 is considered low adherence. Participants will complete the MEDAS at study screening for assessment of eligibility and again at 12 weeks.

### 2.8. Demographic Questionnarie

At baseline only, participants will be asked to complete a series of sociodemographic questions related to age, country of birth, cultural background, level of mobility, smoking status, education, socioeconomic status, current medical conditions, medication and supplement use.

### 2.9. Dietary Protocol

Participants will be randomly allocated to either a 12 week prescribed and individualized ad libitum MedDiet intervention; or a 12 week prescribed and individualized ad libitum healthy eating (HE) dietary approach. Participants randomized to the HE dietary approach will be asked to follow a dietary pattern consistent with the Australian Dietary Guidelines and Australian Guide to Healthy Eating. Specifically, this will include a dietary pattern consisting of wholegrain breads and cereals, vegetables, fruit, low-fat dairy, lean meat, poultry and fish and small servings of unsaturated fats.

The MedDiet intervention will be in line with traditional MedDiet principles yet adapted to suit the dietary and cultural preferences of the Australian population. Notably, the MedDiet intervention will emphasize a high intake and wide variety of plant-based foods (fruits, vegetables, wholegrains, legumes and nuts), liberal and exclusive use of extra virgin olive oil (EVOO), moderate intake of fish and seafood and red wine with a limited or null intake of processed foods, butter and red or processed meat. The specific details for each of the dietary approaches, including target serving sizes, are outlined in Table 2. Nil caloric restriction will be prescribed for either dietary approach, and participants will be asked to maintain their habitual physical activity levels throughout the study duration.

### 2.10. Education and Counselling

All participants will receive individual dietary counselling and education by the study dietitian each fortnight for the duration of the study (5 sessions in total, 30 min in duration each). Both dietary protocols will be presented to participants in the form of a dietary checklist outlining key foods and recommended target serve sizes (daily/weekly) for participants to achieve. Dietary checklists will also be discussed with the study dietitian to clarify serving sizes and address any potential issues or problems with compliance toward the dietary protocol. To further facilitate dietary compliance, all participants (irrespective of their group allocation) will be provided with a suite of written educational resources presented in the form of an information pack developed by the research team. Specifically, the information pack will include a fridge magnet, shopping lists, meal suggestions, recipes, label reading resources, health pamphlet and a pictorial display of the recommended number of serves and serve sizes for all recommended foods. In addition, all participants will receive weekly one-way text messages designed to facilitate behaviour change and provide a health prompt to facilitate dietary compliance.

The provision of education and counselling for all participants will further involve mutually set Specific, Measurable, Achievable, Realistic, Timely (SMART) goal principles and established action plans for achieving these goals. The SMART goals will be individualized and adapted to the capacities of women so that the counselling and the SMART goals are tailored to the health literacy levels of women. Participants will further be asked to assess their level of confidence in executing their goals, how important they feel the goal is for adhering to the dietary intervention and provide self-reported feedback of goal implementation. In addition to the provision of counselling and education toward the dietary protocols, each of the fortnightly study visits will be structured around central themes related to each of the dietary protocols. Specifically, this will include exploring cooking skills and availability of cooking equipment, convenience meals and options for eating out (week 2) and building skills around goal setting, problem-solving and self-efficacy (week 4). The following consultations will provide education on the specific components of each of the dietary protocols, including dietary fats and label reading (week 6), fruit and vegetable intake (week 8), and intake of wholegrain breads and cereals (week 10). Further, we developed an advisory committee consisting of an Obstetrician and Gynecologist, Endocrinologist, Dietitian and a consumer representative who provided additional strategic advice related to the development of the study protocol, and the development and presentation of educational resources to ensure maximum engagement with the target population.

### 2.11. Digital Messaging

To further promote study engagement and facilitate dietary compliance, all participants, independent of their group allocation, will receive health prompts in the form of weekly one-way text messages. Text message support has been shown to be an acceptable and positive approach to facilitate adherence to multiple lifestyle behaviour changes in patients with coronary heart disease [48]. Moreover, such techniques have been identified as beneficial to promote adherence to healthy eating principles [49], including adherence to a MedDiet [50] and to improve diet quality in adults with T2DM [51] and CVD [52]. Nevertheless, it has previously been identified that text message support is more suitable as an ‘add-on’ to a behavioural and lifestyle intervention led by medical professionals [53]. Text messages will be standardized for each dietary protocol such that each participant will receive the same text messages as other participants in their treatment allocation. Moreover, the messages have been developed to align and address components of the COM-B model [54].

### 2.12. Acceptability and Feasibility of the MedDiet Intervention: Theoretical Frameworks into Practice

**Figure 2:**
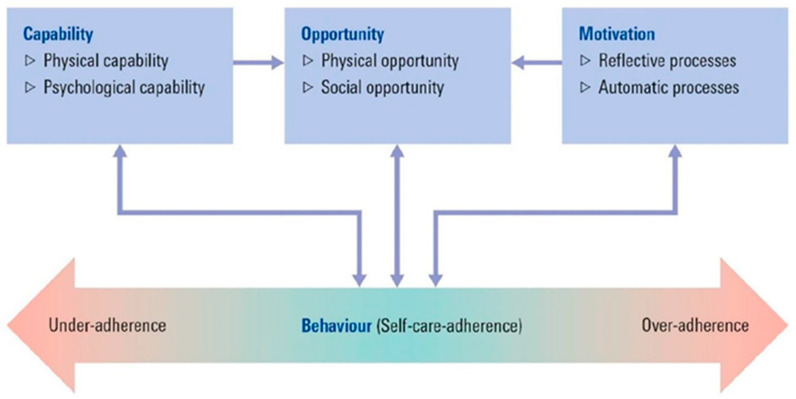
The capability, opportunity and motivation behaviour (COM-B) model and its components [69].

Understanding the relationship between behaviour change techniques (BCTs) and mechanisms of action (i.e., the processes through which BCTs influence behaviour), helps inform the development of behaviour change interventions [55,56,57]. Specifically, in the context of the present study, using behavioural theory to identify the determinants of behaviour change can increase the likelihood of the intervention being effective [58]. The Capability, Opportunity, Motivation (COM-B) model of behaviour [56] in combination with the BCT Taxonomy [59] will be employed in the development and evaluation of the MedDiet intervention (Table 3). Specifically, the 93-item hierarchical BCTs have been developed as a method for specifying, evaluating, and implementing behavioural change interventions [59]. In the proposed study intervention, we have utilized specific BCT to help encourage self-management as they are important components to lifestyle interventions to help individuals initiate [60] and sustain the healthy lifestyle behaviours [61]. Further BCTs were also included to facilitate dietary behaviour change and address barriers for dietary adherence [62,63,64]. The COM-B model suggests that for any behaviour to occur, a person must possess the psychological and physical capability to perform the intended behaviour; the physical and social opportunity to engage in the behaviour and must be motivated to do so. In order to capture important distinctions, these three components are further divided into two subcategories (Figure 2). ‘Capability’ is subdivided into psychological capability and physical capability, ‘opportunity’ is subdivided into physical opportunity and social opportunity and ‘motivation’ is subdivided into reflective processes and automatic processes. Importantly, the use of the COM-B model for the development of behaviour change interventions comprises a systematic, theory-based approach that is aligned with the Medical Research Council recommendations when developing an intervention [65]. Furthermore, the COM-B model has also been used for the identification of barriers and enablers related to adherence toward lifestyle modification interventions, such as diet and physical activity, including women with PCOS [66,67,68].

### 2.13. Participant Interveiws

Participants randomized to receive the MedDiet intervention will also participate in a face-to-face individual semi-structured interview with an Accredited Practicing Dietitian (NS) who has previous qualitative experience and training, at the completion of the baseline and week 12 assessments. Interviews will be audio-recorded, and any noteworthy context or ideas identified by the researcher will be annotated in the individual participant record at the end of the assessment. No other researchers will be present during the time of the interviews. Each interview will consist of six opened-ended questions related to capability, opportunity, motivation and behaviour (COM-B) toward adopting a MedDiet and is expected to take approximately 30–45 min to complete (Table 4). Moreover, the same study participants will be asked a series of questions answered using a Likert scale, to assess their level of confidence in their ability to adhere to a MedDiet (Table 5). Specifically, participants will be asked to rate themselves in relation to eight explicit statements. Scores range from one, indicating very high, to five, indicating very low or no confidence. Upon completion of the study (week 12 assessment), participants will be asked to repeat the exercise, providing a rating of their level of confidence in their ability to adhere to a MedDiet compared with their feelings when assessed at baseline. The design of the semi-structured interview questions and the Likert scales were guided by the COM-B framework [54] (See Table 4 and Table 5).

Furthermore, at the week 12 assessment, we will also assess the feasibility of the MedDiet intervention, where participants will be asked a further six open-ended questions to assess participants’ attitudes and opinions on the educational resources provided throughout the intervention, suggested improvements, and their feelings towards following a MedDiet, independent of caloric restriction and/or weight loss (Table 6). Moreover, participants will also be asked a series of Likert scale questions to indicate their level of agreement to 18 explicit statements regarding the provision of the educational resources and the program delivery (Table 7). Specifically, participants will be asked to indicate their agreement with 18 statements, with a score ranging from one indicating a strong agreement to five indicating strong disagreement. The open-ended questions and Likert scales have been developed based on the feasibility framework published by Bowen et al. [70]. As such, the framework proposes that there are eight general areas of focus addressed by feasibility studies—acceptability, demand, implementation, practicality, adaptation, integration, expansion and limited-efficacy testing [70].

## 3. Analyses of Results

All continuous variables will be presented as the mean (±SD) or the median (IQR) with categorical variables presented as frequencies or percentages. For the efficacy component of the study, paired *t*-tests will be used to identify whether outcome measures change over time. After adjusting for potential covariates, analysis of covariance (ANCOVA) will be applied to identify differences between diet groups over time, with the dietary groups as the independent variable and outcome parameters (primary and secondary outcomes) as the dependent variable. All quantitative analyses will be performed using Statistical Package for the Social Sciences (SPSS) for Windows 26.0 software (IBM Corp., Armonk, NY, USA), with statistical significance set at *p* ≤ 0.05. Primary analyses will be carried out with results analysed based on the “intention to treat” protocol. The required sample size for the proposed study is powered based on change in HOMA-IR (primary outcome of the study) as reported by Ryan et al. [71]. For the proposed study, it was estimated that a total of *n*  =  32 participants would provide 80% power to detect a significant (*p*  <  0.05, 2-sided) change in HOMA-IR by 1.7 ± 0.5. Assuming an attrition rate of 30%, the target sample size for the proposed study will be *n*  =  42 participants (MedDiet intervention *n* = 21; HE dietary approach *n* = 21).

For the acceptability and feasibility component of the study, all semi-structured interviews will be audio-recorded and transcribed verbatim. Data will be analysed through open coding, whereby codes will be iteratively revised and deductively clustered into subthemes and themes according to the framework technique proposed by Richie et al. [72]. These themes will then be mapped against the COM-B constructs. All participant interviews, coding and theme development will be performed by NS. To ensure reliability, successive 10% of the transcripts will be independently coded by another member of the research team until consistent coding occurs. In the event of discrepancies, coded transcripts will be discussed and recoded until a consensus is reached. Analyses of qualitative data will be performed using NVIVO software (QSR International, Melbourne, Australia), version 12.

## 4. Discussion

This study presents a novel intervention examining the efficacy of an ad libitum MedDiet intervention on cardiometabolic, hormonal and anthropometric features in overweight and obese women with PCOS. To the best of our knowledge, this will be the first study of its kind to examine the potential benefits of a MedDiet intervention in women with PCOS, without the need for caloric restriction. Typically, dietary interventions targeted for overweight and obese women with PCOS are designed to create a caloric deficit to elicit weight loss. However, achieving caloric restriction and weight loss targets has previously been shown to be challenging compared to women without PCOS [16].

In a secondary analysis of four clinical trials assessing the effect of weight loss on anthropometric, reproductive, metabolic and/or psychological outcomes in women with PCOS (*n* = 221), Moran et al. [73] reported that although 63% of women achieved clinically significant weight loss of ≥5% over a 2–8 month intervention period, up to 47% of women were lost to attrition, which is also consistent with previous literature [74,75,76]. Given that IR is a prominent clinical feature in the pathophysiology of PCOS, a dietary approach based on key principles of a MedDiet, without the need for weight loss, may indeed represent a novel dietary intervention for women with and without obesity. In a case–control observational study of Italian women (*n* = 112 with PCOS; *n* = 112 controls), greater adherence to a MedDiet was inversely associated with IR and hyperandrogenemia [77]. Moreover, the investigators further reported marked differences in the habitual dietary behaviours between the two groups, whereby women with PCOS consumed less EVOO, legumes, fish/seafood, and nuts compared to women without PCOS. In corroboration with the aforementioned study, Cutillas-Tolín et al. [78] reported that adherence to a MedDiet was protective against clinical phenotypic features of PCOS, namely hyperandrogenism and oligo-anovulation. Moreover. Barrea et al. [79] recently reported that poor adherence to a MedDiet was associated with a ‘metabolically unhealthy obesity’ phenotype in women with PCOS, as defined through evaluation of endocrine-metabolic profiles, inflammatory status, cardiometabolic indices and body composition parameters. Nevertheless, the absence of well-designed clinical trials exploring the efficacy of a MedDiet intervention in women with PCOS represents a notable gap in the research literature.

An important strength of our study is to explore the acceptability and feasibility of a MedDiet intervention in a non-Mediterranean population. Although the MedDiet is one of the most widely reported dietary patterns, much of the evidence from robust clinical trials has previously been conducted in Mediterranean populations [80,81], with few Australian clinical trials reporting on the efficacy and acceptability of a MedDiet intervention [82]. Nevertheless, despite its traditional origins, the translation of a MedDiet to non-Mediterranean populations is indeed appealing. Two previous feasibility studies delivering a MedDiet intervention reported that participants felt confident in their capabilities of long-term adherence to the dietary pattern [50,83]. However, our previous work [64] showed that while the majority (75%, *n* = 606) of Australian consumers perceived health benefits associated with adherence to the MedDiet, they also identified a number of barriers that would limit their ability to adhere to the diet, including knowledge, motivation, affordability, time and suitability. To the best of our knowledge, this will be the first study to explore the acceptability and feasibility of a MedDiet intervention in women with PCOS. Importantly, these data will provide a better understanding of the unique barriers and enablers to dietary change from the perspectives of women with PCOS. As such, use of COM-B model will aid in a deeper understanding as to why women with PCOS adhere and/or are unable to adhere when following a MedDiet intervention, allowing tailoring of future interventions around these barriers and enablers to enhance uptake of future interventions.

Another important strength of this study is that the delivery of the MedDiet intervention is based on an evidenced based theoretical framework. There is evidence to suggest that lifestyle change interventions that are informed by evidence-based behaviour change principles are more effective than those without [84,85]. Importantly, the COM-B model provides a systematic and transparent framework of identifying target behaviours and behaviour change techniques that are deemed to be most effective in behaviour change [69]. Specifically, patient-centered goal setting and improving self-efficacy through the provision of personalized one-on-one counselling and professional support has previously been identified a major facilitator to lifestyle reform amongst women with PCOS [67,86,87,88]. Nevertheless, there is a paucity of studies in PCOS that have applied theoretical frameworks and/or clearly defined and described behavioural change components, making the generalizability and transferability of study findings challenging. Importantly, in the proposed study, the information gained from the structured interviews with PCOS participants will further provide insights that are indeed unique to women with PCOS, including the BCTs used as part of the BCT taxonomy. Lastly, ongoing consultation with potential key stakeholders, including end users and healthcare providers, is an important strength of this study. As such, understanding the experiences and needs of women with PCOS and their healthcare providers in the delivery of evidence-based guidelines is an important step in the development of a theory-based PCOS lifestyle program and successful implementation of behaviour change strategies in order to achieve real-world impact [89].

Whilst the proposed study has several strengths, it also important to acknowledge its limitations. Specifically, we had a lack of available funds to collect additional cardiometabolic parameters including lipids and markers of systemic inflammation. Moreover, we have not included an assessment of psychological distress, in particular mood, depression and anxiety, all of which are particularly pertinent in women with PCOS. Given that this is intended to be a small study with very specific study aims and objectives, prioritization of data collection has been limited to cardiometabolic, hormonal and anthropometric features of PCOS. Importantly, our results will provide preliminary evidence which can be further explored using longer-term and adequately powered multimodal clinical trials investigating the feasibility of a MedDiet intervention, coupled with additional lifestyle related behaviour changes such as physical activity and mindfulness, on changes in body composition, cardiometabolic parameters, inflammation, menstrual cyclicity, fertility, depressive symptomology, and quality of life in overweight and obese women with PCOS.

Importantly, results from this study will offer an evidence-based dietary approach for the management of PCOS. As a secondary outcome of the study, we will also identify the acceptability and feasibility of a MedDiet in women with PCOS. Being able to identify and address potential barriers as well as gain useful insight into specific BCTs is an important step for informing effective and acceptable dietary interventions specific to the management of PCOS that are useful and effective in clinical practice, which is indeed an important strength of the proposed study. Future publications will detail major outcomes from this proposed study. Moreover, we will also communicate our findings more broadly to nutrition professionals and other health-care professionals including General Practitioners, Endocrinologists and Obstetricians, Gynecologists and Allied Health professionals. Specifically, we expect that our findings will also contribute to the future development of consumer and health professional resources and health professional training around the principles of a MedDiet. Lastly, we also expect that our findings could contribute to the evidence base for future iterations of the lifestyle management section of the international evidence-based guidelines for the assessment and management of PCOS [22], with a particular focus on specific therapeutic dietary interventions.

## Figures and Tables

**Table 1:** Participant inclusion and exclusion criteria.

**Inclusion criteria**	Diagnosis of polycystic ovary syndrome defined against the Rotterdam criteria BMI ≥25 kg/m^2^ Aged ≥18 and ≤45 years English speaking
**Exclusion criteria**	Pregnancy Type 1 and 2 diabetes Cushing’s syndrome Hypothyroidism Androgen tumours Active cancer Taking hormonal contraceptives or insulin-sensitising medications within 3 months of the trial start date Mediterranean Diet Adherence Score (MEDAS) ≥10

**Table 2:** Recommended foods and serve sizes for the two dietary protocols.

	Healthy Eating Dietary Approach	Mediterranean Diet Intervention
**Grains**	Include 6 serves daily, ensuring mostly of a wholegrain variety	Include 4–6 serves daily of wholegrains
**Vegetables**	Include 5 serves of vegetables (approximately 375 g), including different types and colours each day. Can include legumes	Include 5–6 serves daily, ensuring vegetables are the central part of the dish. Include a variety of types and colours at every meal. Choose seasonal where possible
**Fruit**	Include 2 serves daily (approximately 300 g)	Include 2–3 serves daily. Choose seasonal when possible
**Legumes**	Include often. Serves are counted toward vegetables and meat/meat alternative recommendations	Include 3 serves weekly
**Fish and Seafood**	Include often	Include 2–3 serves weekly
**Red Meat**	Include lean options regularly, a maximum of 455 g per week	Limit to a maximum of 100 g per week of lean options
**Poultry**	Include often	Include 100–150 g, 1–3 times per week
**Dairy**	Include 2 ½ serves daily and select low-fat options	Include 200 g Greek yoghurt 3 times per week
**Eggs**	Include up to 7 eggs weekly	Include 4–6 eggs weekly
**Nuts**	Limit to small amounts	Include a 30 g serve 3 times per week
**Fat**	Limit to small amounts of polyunsaturated and monounsaturated fats	Include liberal amounts of extra virgin olive oil, aiming for 1–4 tablespoons daily
**Alcohol**	Limit and include 2 alcohol-free days per week	Include up to 200 mL of red wine (2 standard drinks) with meals (only for those who ordinarily consume alcohol), include 2 alcohol-free days per week
**Discretionary foods**	Limit foods high in saturated fat, salt, sugar and refined carbohydrates	Limit intake of sweets, pastries and soft drinks to special occasions only

**Table 3:** Dietary intervention components aligned with behaviour change techniques [59].

Intervention Components	Behaviour Change Techniques
Individualized dietary consults	1.1 Goal setting 1.4 Action planning 1.5 Review behaviour goal 4.1 Instructions on how to perform a behaviour
Resource pack(fridge magnet, shopping lists, pictorial dietary approach guidelines, health pamphlet)	7.1 Prompts/cues 12.5 Adding objects to the environment 5.1 Information about health consequences 4.1 Instructions on how to perform a behaviour
Dietary education sessions	4.1 Instructions on how to perform a behaviour 1.2 Problem solving
Digital messages	7.1 Prompts/cues

**Table 4:** Semi-structured interview questions to identify the feasibility of a MedDiet intervention.

	Question	COM-B
**1.**	Could you describe to me what you think a Mediterranean diet is? *Prompt: which foods/beverages do you think are included?*	Capability (Psychological)
**2.**	What factors or circumstances would help (make it easier) you to follow a Mediterranean Diet?	Motivation (Reflective)
**3.**	What factors or circumstances would make it harder for you to follow a Mediterranean Diet?	Motivation (Reflective)
**4.**	What skills do you think you will need to follow this diet?	Motivation (Reflective)
**5.**	In what ways do you think following a Mediterranean diet could affect your health/lifestyle?	Motivation (Reflective)
**6.**	How do you feel about following a Mediterranean diet? *Prompt: is it something that you enjoy? Do you look forward to it?*	Motivation (Automatic)

**Table 5:** Likert scale to evaluate participant confidence in MedDiet adherence.

	Statement	COM-B
**1.**	How would you rate your knowledge of what foods are part of a Mediterranean diet?	Capability (Psychological)
**2.**	How would you rate your confidence to prepare/cook the food included in a Mediterranean diet?	Capability (Physical)
**3.**	How would you rate your confidence toward having the time to cook/eat a Mediterranean diet?	Opportunity (Physical)
**4.**	How would you rate your ability to afford foods that are required for a Mediterranean diet?	Opportunity (Physical)
**5.**	How would you rate your access to the foods that are required for a Mediterranean diet?	Opportunity (Physical)
**6.**	How would you rate the acceptability of a Mediterranean diet by your friends or family?	Motivation (Reflective)
**7.**	How would you rate your ability to adhere to a Mediterranean diet?	Motivation (Reflective)
**8.**	How would you rate your intention to follow a Mediterranean diet?	Motivation (Reflective)

**Table 6:** Assessment of dietary intervention resources and deliver.

	Open-Ended Questions	Bowen Framework
**1.**	What was your view on the education resources?	Acceptability
**2.**	Which resource/s was the most helpful?	Demand
**3.**	Which resource/s was the least helpful?	Demand
**4.**	Do you have any suggested improvements on the delivery of the dietary intervention and/or education resources/materials?	Acceptability
**5.**	How did you feel about following a dietary approach independent of calorie restriction or weight loss? *Prompt: did it make it easier or harder to adhere? Did you feel it would be helpful or not helpful for your health?*	Acceptability
**6.**	Any final comments that were not described in previous questions?	

**Table 7:** Evaluation of intervention resources and program delivery.

	Likert Scale	Bowen
**1.**	I found the education resources **easy** to understand	Acceptability
**2.**	I found the education resources **difficult** to understand	Acceptability
**3.**	I found the education resources **easy** to read	Acceptability
**4.**	I found the education resources **difficult** to read	Acceptability
**5.**	I found the education resources **were** useful	Demand
**6.**	I found the education resources were **not** useful	Demand
**7.**	The education resources made it **easier** to adhere to a Mediterranean diet	Demand
**8.**	The education resources made it **difficult** to adhere to a Mediterranean diet	Demand
**9.**	I would have liked **more** resources	Acceptability
**10.**	I would have liked **less** resources	Acceptability
**11.**	I found the dietary consultations **were** useful	Demand
**12.**	I found the dietary consultations were **not** useful	Demand
**13.**	I found the text messages **were** helpful?	Demand
**14.**	I found the text messages were **not** helpful?	Demand
**15.**	I would have liked less text messages?	Acceptability
**16.**	I would have liked **more** text messages?	Acceptability
**17.**	The text messages were too **long**?	Acceptability
**18.**	The text messages were too **short**?	Acceptability

## Data Availability

Materials described in this paper pertain to the study protocol only.
